# HIV infection and engagement in HIV care cascade among men who have sex with men and transgender women in Kigali, Rwanda: a cross‐sectional study

**DOI:** 10.1002/jia2.25604

**Published:** 2020-10-01

**Authors:** Jean Olivier Twahirwa Rwema, Carrie E Lyons, Sara Herbst, Benjamin Liestman, Julien Nyombayire, Sosthenes Ketende, Amelia Mazzei, Oluwasolape Olawore, Sabin Nsanzimana, Placidie Mugwaneza, Aflodis Kagaba, Patrick S Sullivan, Susan Allen, Etienne Karita, Stefan D Baral

**Affiliations:** ^1^ Department of Epidemiology Key Populations Program Center for Public Health and Human Rights Johns Hopkins Bloomberg School of Public Health Baltimore MD USA; ^2^ Projet San Francisco Kigali Rwanda; ^3^ HIV and AIDS Division Rwanda Biomedical Center Kigali Rwanda; ^4^ Health Development Initiative Kigali Rwanda; ^5^ Emory University Atlanta GA USA

**Keywords:** HIV care continuum, structural determinants, men who have sex with men, Kigali, Rwanda

## Abstract

**Introduction:**

Given intersecting biological, network and structural risks, men who have sex with men (MSM) and transgender women (TGW) consistently have a high burden of HIV. Although MSM are a key population in Rwanda, there are limited epidemiologic data to guide programming. This study aimed to characterize HIV prevalence and care cascade among MSM and TGW in Kigali.

**Methods:**

MSM and TGW ≥ 18 years were recruited using respondent‐driven sampling (RDS) from March–August 2018 in Kigali. Participants underwent a structured interview including measures of individual, network and structural determinants. HIV and sexually transmitted infections (STI) including syphilis, Neisseria gonorrhoea (NG) and Chlamydia trachomatis (CT) were tested. Viral load was measured for MSM living with HIV. Robust Poisson regression was used to characterize the determinants of HIV infection and engagement in the HIV treatment cascade.

**Results:**

A total of 736 participants were enrolled. The mean age was 27 years (range:18 to 68) and 14% (106) were TGW. HIV prevalence was 10% (RDS‐adjusted: 9.2% (95% CI: 6.4 to 12.1)). Unadjusted prevalence of any STI was 20% (147); syphilis: 5.7% (42); CT: 9.1% (67) and NG: 8.8% (65). Anticipated (41%), perceived (36%) and enacted stigmas (45%) were common and higher among TGW (*p* < 0.001). In multivariable RDS adjusted analysis, higher age (aPR: 1.08 (95% CI: 1.05 to 1.12)) and ever having sex with women (aPR: 3.39 (95% CI: 1.31 to 8.72)) were positively associated with prevalent HIV. Being circumcised (aPR: 0.52 (95% CI: 0.28 to 0.9)) was negatively associated with prevalent HIV infection.

Overall, 61% (45/74) of respondents reported knowing their HIV‐positive status. Among these, 98% (44/45) reported antiretroviral therapy use (ART); 75% (33/44) were virally suppressed using a cut‐off of <200 copies/mL. Of the 29 participants who did not report any previous HIV diagnosis or ART use, 38% (11/29) were virally suppressed. Cumulatively, 59% (44/74) of all participants living with HIV were virally suppressed.

**Conclusions:**

These data show a high burden of HIV among MSM/TGW in Kigali, Rwanda. Bisexual concurrency was common and associated with prevalent HIV infection, demonstrating the need of comprehensive screening for all sexual practices and preferences in the provision of comprehensive HIV prevention services in Rwanda. Viral suppression was below the UNAIDS target suggesting poor adherence and potential ART resistance. There is a need for adherence support, screening for primary and secondary ART resistance and stigma mitigation interventions to optimize HIV‐related outcomes for MSM in Rwanda.

## 1 INTRODUCTION

Traditionally, HIV epidemics in eastern and southern African countries have been described as generalized epidemics. This definition is predicated on an assumption that HIV risks are homogenous among people living in those settings (1). More recent studies across Africa have consistently shown that key populations including men who have sex with men (MSM) and transgender women (TGW) have higher HIV prevalence and incidence compared with other adults of reproductive age (2, 3, 4, 5, 6). Data from eight countries across sub‐Saharan Africa (SSA) found a pooled HIV prevalence of 14% among cisgender MSM and 25% among TGW (7). The higher risk of HIV infection among MSM and TGW is due in part to individual‐level risks including condomless anal sex among serodiscordant and viraemic partners, and higher prevalence of sexually transmitted infections (STIs) (3, 8). Individual HIV risks are contextualized by community‐level and structural factors including stigma, sexual and physical violence and exposure to human rights violations, which influence individual HIV risk factors (2, 3). Furthermore, structural determinants increase HIV risk by limiting the uptake and provision of HIV prevention and treatment services for MSM (9). Consequently, MSM generally have lower engagement in HIV prevention and treatment services and poorer outcomes compared to other adults in SSA (10, 11).

In Rwanda, the overall HIV prevalence is 3% among reproductive‐aged adults and 2.2% among men (12). According to the 2019 global AIDS update, Rwanda can achieve the UNAIDS 90‐90‐90 targets (13). Despite a successful national HIV programme, there are limited data on the epidemiology of HIV and STIs among MSM and TGW in Rwanda. In 2011, Chapman et al. discussed the “lack of clear HIV policy for MSM due in part to the denial of existence of MSM in Rwanda by some HIV experts” (14). This study did not estimate HIV prevalence, but reported sexual practices that would predispose MSM to HIV and STI risks including condomless sex and frequent sex work (14). A 2015 study estimated an HIV prevalence among MSM (4.8%) higher than the national average (15). Furthermore, these studies described existence of bisexual partnerships among Rwandan MSM similar to settings around the world where same‐sex practices are stigmatized and sustained relationships are challenged (16, 17, 18). However, whether MSM who have sex with both men and women in Rwanda have differential HIV and STIs risks remains unknown. Additionally, the burden of STIs has mostly relied on self‐reported data and engagement in HIV care cascade of MSM and TGW is not well characterized. Finally, though same‐sex practices are not explicitly criminalized in Rwanda, stigma, sexual and physical violence against MSM have been reported but have not been comprehensively investigated as determinants of HIV infection among Rwandan MSM and TGW (14, 18).

This study was conducted to address these knowledge gaps and inform the content and implementation of HIV prevention and treatment services for MSM and TGW in Rwanda.

## 2 METHODS

### 2.1 Study population and procedures

This was a cross‐sectional behavioural and biological assessment for MSM in Kigali, Rwanda. Eligible participants were individuals assigned the male sex at birth, aged 18 years or older, who had lived in Kigali for at least three months before the study, and who reported anal sex with a man in the preceding 12 months. Community engagement leveraged existing connections of Projet San Francisco (PSF) and Health Development Initiative (HDI) with 10 MSM and transgender associations in Kigali. These community members were hired as study staff, were involved in the translation of the survey instrument in Kinyarwanda to ensure the use of appropriate local language, community mobilization and selection of the study site.

Recruitment occurred from March‐August 2018 using respondent‐driven sampling (RDS), a method used to recruit marginalized populations (19). Two seeds, MSM who were well‐connected in the MSM community were recruited to initiate recruitment. A third seed was recruited during the study to enhance recruitment of older participants. Efforts were made to select individuals with different characteristics to maximize sample heterogeneity.

Study procedures were performed at the study site and included a structured interview and biological testing. Trained nurse counsellors conducted the eligibility screening using a questionnaire and obtained signed informed consent (Table S1). The interview guide was based on a modified social ecological model to assess individual‐, community‐, network‐ and structural‐level risks of HIV risk among MSM (20). To protect participant’s privacy, no personal identifying information was collected.

HIV status was determined by serial rapid testing. HIV screening used Alere HIV Combo – Determine (Alere, Inc, Waltham, MA) and confirmatory testing used HIV ½ STAT‐PAK (Medford, NY, USA) as per Rwandan national guidelines. Viral load testing was performed for all participants biologically confirmed to be living with HIV at the National Reference Laboratory of Rwanda (NRL). Syphilis antibodies were screened using rapid plasma reagin (RPR – Santa Coloma, Spain) and screened positive specimens were confirmed using the Treponema Pallidum haemagglutination assay (TPHA – Langdorp, Belgium). Chlamydia trachomatis and Neisseria gonorrhoea were tested using Cepheid GeneXpert platform Xpert CT/NG (Solna, Sweden) on self‐collected urine and rectal swabs. Newly HIV diagnosed participants were referred to a health facility of their choice for ART initiation. Those who tested positive for any STI were treated without charge at the study site according to the Rwanda national guidelines.

Upon completion of study procedures, each participant was given three study coupons to recruit peers into the study. Participants received 3000Frw and the same amount for each eligible participant they referred to the study. This study was approved by the Institutional Review Board of Emory University (IRB00089599) and the Rwanda National Research Ethics committee.

### 2.2 Outcomes

The primary outcome was HIV prevalence. HIV‐positive status was defined as a positive result on both the screening and confirmatory tests. In case of discordant results, samples were sent to NRL for ELISA and adjudication.

Engagement in HIV care and description of the progress to achieve the 90‐90‐90 goals among MSM in Kigali were defined as the proportion of MSM who knew their HIV‐positive status (first 90) before the study test and who were on ART (second 90). Viral suppression (third 90) was based on laboratory testing and was defined as a viral load of <200 copies/mL as per the national guidelines (21).

### 2.3 Covariates of interest

A list of individual and structural determinants of HIV and their hypothesized association with HIV infection among MSM/TGW is in the Figure S1.

### 2.4 Individual determinants

Individual determinants were demographic, biological, behavioural and health‐related factors (Tables 1,2,3). Gender identity was self‐reported using a two‐step instrument and dichotomized into cisgender‐MSM or TGW based on gender assignment at birth and current gender identity (22). Sexual preference was self‐reported and categorized as homosexual, bisexual and heterosexual. Biological and health‐related factors included STI infection, circumcision and mental health status. STI diagnosis was determined by biological testing and circumcision was self‐reported. Depression was assessed using the Patient Health Questionnaire‐9 (PHQ‐9) (23, 24) and categorized using established cut‐points: none (0 to 4), mild (5 to 9), moderate (10 to 14), moderately severe (15 to 19) and severe depression (≥20).

**Table 1:** Demographic characteristics of MSM and TGW enrolled in Kigali, Rwanda 2018 (N = 736)

Characteristic	N	Crude %	RDS‐Adjusted % (95% CI)
Age in years			
18 to 24	335	45.6	48.9 (43.8 to 54.1)
25 to 34	295	40.0	35.9 (30.9 to 41.1)
>35	106	14.4	15.2 (11.5 to 18.5)
Education			
Never attended school	24	3.3	4.5 (2.0 to 6.9)
Primary	185	25.2	30.3 (25.6 to 35.1)
Some secondary	245	33.2	31.5 (26.7 to 36.4)
Secondary or above	282	38.3	33.7 (28.7 to 38.6)
Marital status			
Single/Never married	653	88.7	87.4 (83.8 ‐ 90.9)
Cohabitating with male partner	27	3.7	3.8 (1.7 ‐ 5.8)
Cohabitating/married with female partner	24	3.3	3.3 (1.4 ‐ 5.2)
Divorced/Separated/Widow	32	4.3	5.5 (2.9 ‐ 8.1)
Occupation			
Unemployed	130	17.6	15.1 (11.4 ‐ 18.8)
Student	89	12.1	12.1 (8.4 ‐ 15.8)
Employed/Self employed	517	70.3	72.8 (68.1 ‐ 77.6)
**Monthly Income (Frw)**			
Less than 50,000	482	65.5	70.3 (65.5 ‐ 75.1)
50,000 to 100,000	178	24.2	23.6 (18.8 ‐ 27.9)
Over 100,000	76	10.3	6.3 (4.3 ‐ 8.3)
**Self‐reported gender identity**			
Cisgender Men who Sex with Men	630	85.6	89.4 (86.4 to 92.2)
Transgender	106	14.4	10.6 (7.8 to 13.5)
Self‐reported sexual preferences			
Gay or Homosexual	475	64.6	58.1 (52.8 ‐ 63.4)
Bisexual	227	30.8	35.6 (30.4 ‐ 40.8)
Heterosexual	34	4.6	6.3 (3.7 ‐ 8.8)
Sex with women			
Never	187	25.4	25.6 (20.7 ‐ 30.4)
Yes, but not in the last 12 months	279	37.9	36.7 (31.7 ‐ 41.6)
Yes, in the last 12 months	270	36.7	37.7 (32.9 ‐ 42.6)
Engagement in sex work			
Never	453	61.7	68.9 (64.3 ‐ 73.7)
Ever provided sex acts in exchange for money	171	23.2	22.0 (17.8 ‐ 26.2)
Main source of income in the previous year	112	15.1	9.0 (6.5 ‐ 11.5)

RDS, respondent‐driven sampling.

**Table 2:** Comparison of men who report sex with men only and men who report sex with both men and women on key sociodemographic, biological, behavioural and HIV/STI outcomes Kigali, Rwanda 2018

	MSMO % (N)	MSMW % (N)	*p* value
Sociodemographic characteristics			
Age in years			
18 to 24	49.7 (93)	44.3 (243)	**0.033**
25 to 34	41.7 (78)	39.5 (217)	
Over 35	8.6 (16)	16.2 (89)	
Education			
Primary level or less	28.3 (53)	28.6 (157)	0.991
Some secondary	33.7 (63)	33.2 (182)	
Secondary or above	38 (71)	38.2 (210)	
Marital status			
Single/Never married	93.6 (175)	87.1 (478)	**0.000**
Cohabitating with male partner	6.4 (12)	2.7 (15)	
Cohabitating/married with female partner	0 (0)	4.4 (24)	
Divorced/Separated/Widow	0 (0)	5.8 (32)	
Monthly income (Frw)			
Less than 50,000	66.8 (125)	65 (356)	0.640
Over 50,000	33.2 (62)	35.0 (192)	
Gender identity			
Cisgender Men who have Sex with Men	74.3 (139)	89.4 (491)	**0.0001**
Transgender women	25.7 (48)	10.6 (58)	
Self‐reported sexual preference			
Gay or Homosexual	86.6 (162)	57.0 (313)	**0.0001**
Bisexual	12.8 (24)	37.0 (203)	
Heterosexual	0.5 (1)	6.0 (33)	
Biological			
Circumcision			
No	24.6 (46)	20.7 (114)	0.272
Yes	75.4 (141)	79.3 (435)	
**Behavioural**			
Age of first sex with male			
Before 19 years	67.4 (126)	49.1 (269)	**0.0001**
19 to 22 years	21.9 (41)	22.8 (125)	
Over 22 years	10.7 (20)	28.1 (154)	
Number of regular sexual partners in the last month			
None	31.2 (58)	45.9 (252)	**0.001**
0ne to two	48.4 (90)	32.6 (179)	
Two to three	9.7 (18)	10.2 (56)	
Over three	10.7 (20)	11.3 (62)	
Number of casual sexual partners in the last month			
None	60.4 (113)	48.8 (267)	**0.018**
One to three	27.8 (52)	38.2 (209)	
Over three	11.8 (22)	12.9 (71)	
Sexual practices with regular male partners			
Insertive anal sex			
Never	17.7 (27)	4.2 (17)	**0.0001**
Ever	82.3 (126)	95.8 (384)	
Receptive anal sex			
Never	34.6 (53)	37.4 (150)	0.546
Ever	65.4 (100)	62.6 (251)	
Sexual practices with casual male partners			
Insertive anal sex			
Never	16.3 (24)	5.6 (27)	**0.0001**
Ever	83.7 (123)	94.4 (455)	
Receptive anal sex			
Never	32.6 (48)	39.2 (189)	0.151
Ever	67.4 (99)	60.8 (293)	
Alcohol use			
Nonhazardous alcohol use	27.8 (52)	22.6 (124)	0.148
Hazardous alcohol use	72.2 (135)	77.4 (425)	
HIV and sexually transmitted infections‐related outcomes			
**HIV infection**			
Negative	93.5 (174)	88.7 (487)	**0.058**
Positive	6.5 (12)	11.3 (62)	
Virally suppressed (based on < 200 copies/mL)			
No	50.0 (6)	37.7 (23)	0.426
Yes	50.0 (6)	62.3 (38)	
STI diagnosis			
Negative	80.7 (151)	79.8 (438)	0.775
Positive	19.3 (36)	20.2 (111)	
Gonorrhoea			
Negative	91.4 (170)	91.1 (499)	0.88
Positive	8.6 (16)	8.9 (49)	
Chlamydia			
Negative	92.5 (172)	90.3 (495)	0.380
Positive	7.5 (14)	9.7 (53)	
Syphilis			
Negative	94.1 (175)	94.3 (518)	0.892
Positive	5.9 (11)	5.6 (549)	

The bold values are variables that were statistically significantly different among participants who report sex with women and those who did not based on the chi‐square p values. The cutoff of significance was *p* < 0.05.

MSMO, men who have sex with men only; MSMW, men who have sex with men and women.

**Table 3:** Individual and structural determinants of HIV infection among MSM and TGW in Kigali, Rwanda

	N	%	Unadjusted PR with 95%CI	*p* value	Adjusted PR with [95%CI]	*p* value	RDS adjusted PR [95%CI]	*p* value
*Sociodemographic characteristics*								
Age in years								
Age (from 18 years of age)	736		**1.06 (1.05 to 1.07)**	**0.0001**	**1.08 (1.06 to 1.12)**	**0.0001**	**1.08 (1.05 to 1.12)**	**0.0001**
District of residence								
Gasabo	89	12.2	Ref	Ref	Ref		Ref	Ref
Kicukiro	162	21.9	0.59 (0.27 to 1.30)	0.196	0.57 (0.26 to 1.25)	0.163	0.96 (0.32 to 2.89)	0.950
Nyarugenge	485	65.9	0.86 (0.46 to 1.57)	0.605	0.72 (0.41 to 1.25)	0.240	1.15 (0.52 to 2.58)	0.725
Education								
Primary level or less	210	28.5	Ref	Ref	Ref		Ref	Ref
Some secondary	244	33.2	**0.41 (0.23 to 0.70)**	**0.001**	0.93 (0.52 to 1.65)	0.806	0.76 (0.33 to 1.75)	0.528
Secondary or above	282	38.3	**0.43 (0.26 to 0.72)**	**0.001**	1.28 (0.65 to 2.51)	0.479	2.01 (0.82 to 4.90)	0.124
Marital status								
Single/Never married	653	88.7	Ref	Ref	Ref		Ref	Ref
Cohabitating with male partner	27	3.7	0.46 (0.06 to 3.18)	0.429	0.41 (0.05 to 3.01)	0.380	0.41 (0.04 to 3.86)	0.440
Cohabitating/married with female partner	24	3.3	**3.46 (1.76 to 6.79)**	**0.0001**	0.54 (0.21 to 1.35)	0.188	0.35 (0.11 to 1.23)	0.104
Divorced/Separated/Widow	32	4.3	**4.08 (2.37 to 7.02)**	**0.0001**	0.85 (0.41 to 1.76)	0.661	0.97 (0.38 to 2.47)	0.950
Monthly Income (Frw)								
Less than 50,000	481	65.4	Ref	Ref	Ref	Ref	Ref	Ref
Over 50,000	254	34.6	**0.61 (0.36 to 1.01)**	**0.056**	**0.56 (0.33 to 0.96)**	**0.036**	**0.36 (0.16 to 0.82)**	**0.015**
Self‐reported gender identity								
Cis MSM	630	85.6	Ref	Ref				
Transgender woman	106	14.4	0.92 (0.49 to 1.75)	0.819				
Self‐reported sexual preference								
Gay or Homosexual	475	64.6	Ref	Ref				
Bisexual	227	30.8	1.03 (0.65 to 1.63)	0.91				
Heterosexual	34	4.6	0.29 (0.04 to 2.07)	0.219				
Biological and other health‐related factors								
STI diagnosis								
No	589	80.1	Ref	Ref	Ref	Ref	Ref	Ref
Yes	147	19.9	**1.81 (1.14 to 2.86)**	**0.011**	**1.45 (0.95 to 2.19)**	**0.081**	**1.56 (0.94 to 2.58)**	**0.08**
Mental health								
Not depressed	475	64.6						
Mild depression	194	26.3	1.17 (0.72 to 1.84)	0.514				
Moderate depression	48	6.5	0.86 (0.32 to 2.29)	0.767				
Moderately severe depression	12	1.6	0.86 (0.13 to 5.7)	0.878				
Severe major	7	1	1.72 (0.28 to 10.5)	0.555				
Circumcision								
No	161	21.8	Ref	Ref	Ref	Ref	Ref	Ref
Yes	575	78.2	**0.43 (0.28 to 0.67)**	**0.0001**	0.74 (0.46 to 1.21)	0.229	**0.52 (0.28 to 0.97)**	**0.041**
Behavioural								
Age of first sex with male								
Before 19 years	396	53.8	Ref	Ref	Ref	Ref	Ref	
19 to 22 years	166	22.6	1.46 (0.81 to 2.66)	0.21	1.13 (0.65 to 1.95)	0.665	0.90 (0.39 to 2.05)	0.805
Over 22 years	174	23.6	**2.79 (1.72 to 4.54)**	**0.0001**	1.28 (0.74 to 2.21)	0.375	0.87 (0.43 to 1.75)	0.688
CCU with male partners in the last six months								
Non‐consistent condom use	642	87.3	Ref	Ref	Ref		Ref	Ref
Consistent condom use	94	12.7	0.60 (0.27 to 1.35)	0.219	0.72 (0.33 to 1.58)	0.419	1.15 (0.53 to 2.48)	0.719
Lubricant use								
Never	95	12.9	Ref	Ref				
Ever used lubricant	641	87.1	0.95 (0.50 to 1.78)	0.87				
Number of regular sexual partners in the last month								
None	310	42.2	Ref	Ref				
One to two	269	36.6	1.28 (0.78 to 2.09)	0.327				
Two to three	74	10.1	1.08 (0.49 to 2.39)	0.838				
Over three	82	11.1	1.26 (0.62 to 2.57)	0.526				
Number of casual sexual partners in the last month								
None	379	51.8	Ref	Ref	Ref	Ref	Ref	Ref
One to three	262	35.6	1.00 (0.62 to 1.63)	0.985	1.25 (0.76 to 2.04)	0.383	0.96 (0.49 to 1.90)	0.910
Over three	93	12.6	1.47 (0.81 to 2.66)	0.201	**2.51 (1.42 to 4.41)**	**0.001**	2.13 (0.86 to 5.30)	0.102
Sex with women								
Never	187	25.5	Ref	Ref	Ref		Ref	Ref
Yes, but not in the last 12 months	279	37.9	**1.88 (1.00 to 3.55)**	**0.049**	1.32 (0.70 to 2.48)	0.386	**3.39 (1.31 to 8.72)**	**0.011**
Yes, in the last 12 months	270	36.6	1.61 (0.89 to 3.08)	0.153	1.33 (0.66 to 2.69)	0.422	**4.3 (1.46 to 12.69)**	**0.008**
Professional sex worker in the last 12 months								
No	624	84.8	Ref	Ref	Ref		Ref	Ref
Yes	112	15.2	0.58 (0.27 to 1.23)	0.159	0.66 (0.31 to 1.44)	0.298	0.89 (0.28 to 2.87)	0.849
Alcohol use								
Nonhazardous alcohol use	175	23.8	Ref	Ref				
Hazardous alcohol use	561	76.2	0.97 (0.58 to 1.60)	0.907				
Structural								
Perceived stigma score								
No	474	64.5	Ref	Ref				
Yes	262	35.5	1.16 (0.75 to 1.81)	0.496				
Anticipated stigma score								
No	438	59.4	Ref	Ref				
Yes	299	40.6	0.94 (0.60 to 1.46)	0.791				
Enacted stigma score								
No	407	55.4	Ref	Ref				
Yes	329	44.6	1.05 (0.68 to 1.62)	0.820				
Access to condom in the last six months								
Easy	501	68.1	Ref	Ref				
Difficult	136	18.5	1.09 (0.62 to 1.93)	0.748				
Not applicable	99	13.4	1.39 (0.79 to 2.49)	0.252				
Access to lubricant in the last six months								
Easy	372	58.1	Ref	Ref				
Difficult	163	25.4	0.72 (0.39 to 1.31)	0.288				
Not applicable^a^	106	16.5	0.90 (0.54 to 1.49)	0.692				

Bold values are for variables that were found to be significantly associated with prevalent HIV infection in the regression analyses.

Cis‐MSM, cisgender men who have sex with men; CCU, consistent condom use; PR, prevalence ratios.

^a^ Participants who did not try to get lubricants in the last six months.

Condom use with male partners was assessed by partner type (regular vs. casual male partners), time scale (last sex and last six months) and anal sex type (receptive or insertive). Consistent condom use (CCU) was defined as having always used condoms during anal sex with male partners in the last six months. For participants who also reported sex with women, frequency and condom use during anal sex with their female partners was assessed. Hazardous alcohol use was screened using the AUDIT‐C (25, 26), with a score of four indicating hazardous alcohol use.

### 2.5 Structural determinants

Structural‐level risks measured stigma in healthcare settings, from family and friends, uniformed officers and in the community. These scales have been validated to assess stigma among MSM in other settings (27). Scores for anticipated stigma, enacted stigma and perceived stigma were dichotomized at 1, where a score of ≥1 for a given stigma scale was considered as experiencing that type of stigma (Table 3). The complete list of the stigma‐related questions and the corresponding scales is in the Table S2. Other structural factors included community level access to condom and condom‐compatible lubricants.

### 2.6 Statistical analyses

Crude numbers and proportions were calculated for all covariates of interest. RDS‐adjusted proportions with 95% confidence intervals (CIs) were calculated for sociodemographic and outcome variables (Tables 1,3) using Stata’s RDS II estimator package (28). Pearson Chi‐squared tests were used to compare participants who have sex with men only (MSMO) and those who report sex with both men and women (MSMW) (Table 2); and Cisgender MSM and TGW (Table S3). Given the high HIV prevalence (>10%), Poisson regression with robust variance estimation was used to model the association between the outcome and covariates as log‐binomial models failed to converge. Bivariable analyses estimated prevalence ratios (PR) and 95%CIs. The final multivariable model used variables associated with the outcome in the bivariable analyses at (*p* < 0.2). Finally, RDS adjustment of the final model was performed to estimate these associations with inference to the Kigali MSM population. All analyses were performed in Stata Version.14.2 (StataCorp, College Station, TX).

## 3 RESULTS

**Figure 1:**
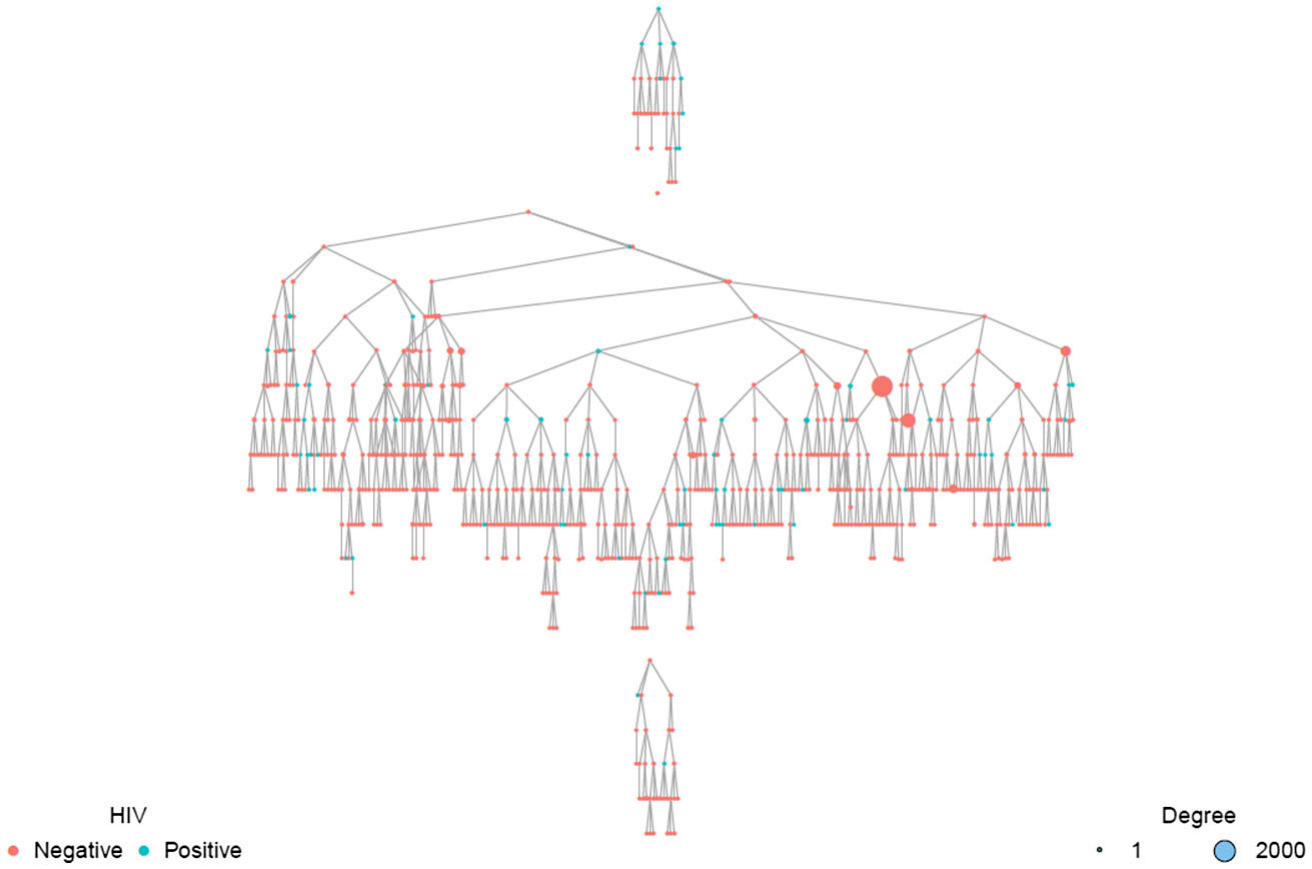
Respondent‐Driven Sampling plot demonstrating recruitment networks and HIV status among men who have sex with men and transgender women in Kigali, Rwanda. The blue nodes indicate participants living with HIV and the red nodes are for HIV‐negative participants. The size of the node is proportional to the network size, participants who know more MSM and TGW have larger nodes compared to participants who are less connected in the MSM/TGW community in Kigali

In total, 738 participants including the three seeds were recruited in 12 recruitment waves (Figure 1). Analyses were restricted to 736 participants with complete data. The mean age was 27 years [range: 18 to 68], 89% were single and 14% were TGW and 31% were bisexual (Table 1).

### 3.1 HIV prevalence

The overall HIV prevalence was 10% (n = 74) RDS‐adjusted 9.2% (95% CI: 6.3 to 12.1). There was no difference in HIV prevalence among cis‐MSM (10.2%) and TGW (9.4%) (*p* = 0.818). HIV prevalence was significantly higher with age: 2% among 18‐ to 24‐year olds, 12% among 25‐ to 34‐year olds and 30% at ≥35 years (*p* < 0.0001).

### 3.2 Individual determinants

One in five participants 20% (n = 147) was diagnosed with at least one STI, 9% (n = 65) had chlamydia, 9% (65) had gonorrhoea and 6% (42) had syphilis. Most participants self‐reported being circumcised (78%). Overall, 26% reported mild depression and 8% reported moderate or worse depression (Table 3).

Overall, 54% reported sexual debut with men before the age of 19. Multiple sexual partnerships in the preceding month included 21% reporting ≥2 regular partners; and 36% reporting 1 to 3 casual male sexual partners. Among participants reporting regular partners, 92% engaged in insertive anal sex and 63% reported receptive anal sex; and CCU in the previous six months was 37% and 33 %, respectively, for insertive and receptive anal sex. Among those reporting casual partners, insertive anal sex was more common than receptive anal sex (92% vs 62%); and CCU was 38% and 36%, respectively, for insertive and receptive anal sex with casual partners.

Three quarters of participants reported ever having sex with women, among whom 49% reported sex with women in the prior year. Overall, 38% of the 226 who reported ever having a regular female partner and 30% of 482 who reported casual female partners reported anal sex with their female partners. Overall, condom use at last anal sex was 50% and 54%, respectively, with regular and casual female partners.

Overall, 87% ever used lubricants during sex, including commercial water‐based lubricants (63%) and petroleum jelly/Vaseline (30%). Among respondents, 38% disclosed ever providing sexual acts in exchange for money and 15% reported sex work as their main source of income in the previous year. Hazardous alcohol use was common (76%).

### 3.3 Structural determinants

Overall, 13% reported ever feeling excluded from family activities and 20% reported discriminatory remarks from family members. Additionally, 28% of participants reported feeling scared to be in public places and 31% experienced verbal harassment. Some participants reported past physical (16%) or sexual (14%) violence. Based on the stigma scales scores, 41% reported anticipated stigma, 36% perceived stigma and 45% reported enacted stigma.

### 3.4 Cisgender and transgender individuals

TGW were different from cisgender MSM in age distribution (*p* = 0.038), marital status (*p* = 0.075) and reported more depressive symptoms (*p* < 0.0001). TGW reported younger age of sexual debut with men (*p* = 0.005), engaged more in sex work (*p* = 0.0001) and reported more lubricant use compared to cisgender MSM (*p* = 0.007). There were no significant differences in condom or alcohol use. TGW experienced higher levels of anticipated, perceived and enacted stigmas (*p* < 0.001) compared to cisgender MSM. There were no differences in HIV and STI outcomes (Table S3).

### 3.5 Men who have sex with men only and men who have sex with both men and women

MSMW differed from those reporting MSMO on several characteristics including age, marital status, gender identity, sexual preference and practices. MSMW reported a higher age of sexual debut with male partners (<0.001), higher number of casual male partners (*p* = 0.018) but lower number of regular partners (*p* = 0.001). MSMW reported more insertive, but similar levels of receptive anal sex with male partners. However, there were no significant differences with condom use with any male partner type or STIs apart from HIV (Table 2).

### 3.6 HIV care cascade

Most participants (91%) reported ever being tested for HIV. Of the 74 participants who tested positive for HIV, 61% (45) reported knowing their status before the study. Of those, 44 (98%) reported being on ART and 33 (75%) were virally suppressed.

**Figure 2:**
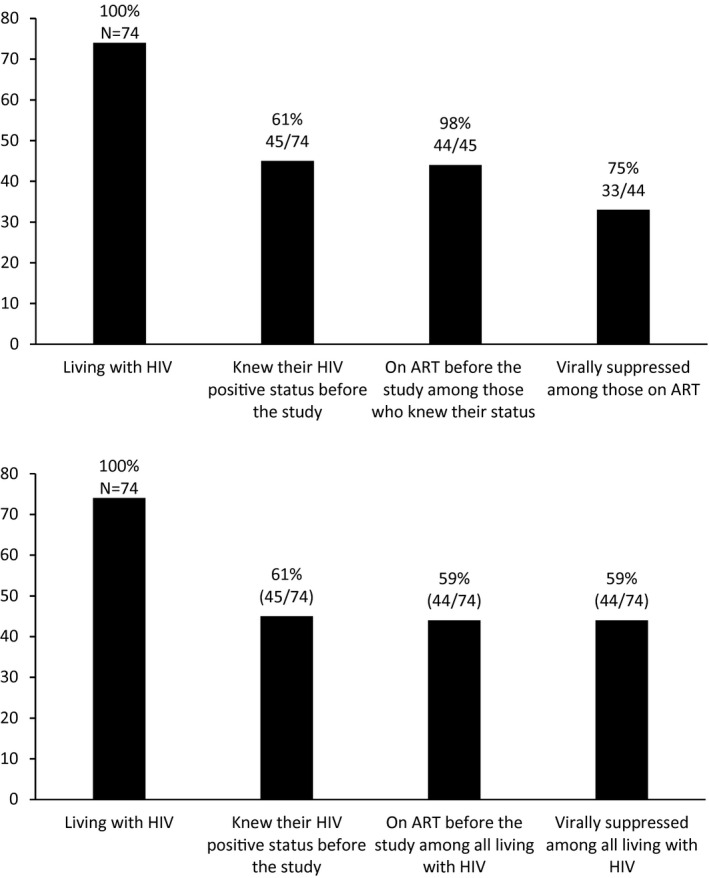
**(A)** HIV care cascade among men who have sex with men and transgender women who knew their HIV‐positive status and were on ART before the study in Kigali, Rwanda. **(B)** HIV care cascade among all men who have sex with men and transgender women living with HIV in Kigali, Rwanda

Among the 29 participants living with HIV, unaware of HIV status prior to the study and reported not being on ART, 38% (11/29) were virally suppressed. Hence, the total proportion of viral suppression among all participants living with HIV was 59% (44/74) (Figure 2).

### 3.7 Factors associated with prevalent HIV infection

In the multivariable RDS adjusted analysis, age (aPR = 1.08;95%:CI 1.05 to 1.12), ever having had sex with women (aPR = 3.39; 95% CI: 1.31 to 8.72) and sex with women in the 12 months preceding the study (aPR = 4.3; 95% CI: 1.46 to 12.69) remained significantly associated with prevalent HIV infection. Having a prevalent STI infection was marginally significantly associated with HIV (aPR = 1.56; 95% CI: 0.94 to 2.58). Being circumcised (aPR = 0.52; 95% CI: 0.28 to 0.97) and having income >50,000 Frw (PR = 0.56; 95% CI: 0.34 to 0.93) was significantly inversely associated with HIV infection. (Table 3).

## 4 DISCUSSION

These data show that MSM and TGW have a higher burden of HIV compared to adult men in Kigali, and across Rwanda (29). They also highlighted factors associated with HIV infection among MSM in Kigali. Older age, low income, STI infection and sex with women were positively associated with HIV infection, whereas circumcision was negatively associated with HIV. These findings are consistent with findings from other studies across SSA demonstrating a high burden of HIV among MSM and TGW (7, 30, 31). Furthermore, HIV prevalence in this study is higher than previously estimated for MSM in Rwanda (15).

This study found a high level of concurrent partnerships with men and women and an increased risk of HIV infection among MSMW. This highlights the need for prevention interventions tailored to the needs of MSMW and screening for all sexual practices in health encounters. Bisexual partnerships are common among MSM across SSA and around the world (16, 32, 33), however, evidence on HIV risk associated with bisexual relationships among MSM is mixed. A study in Mozambique found that MSMW had lower odds of HIV infection compared to MSMO (17). A systematic review focused on China found a higher prevalence of HIV among MSMW compared to MSMO (34), whereas another systematic review focused on Asia found no difference in HIV prevalence between those two groups (32). For Rwanda, it is difficult to conclude the reason that HIV among MSMW was higher based on these cross‐sectional data. This might be explained in part by the differences in age, age of sexual debut, gender identity, sexual preference and practices among MSMW and MSMO. MSMW were older and reported more male casual partners in the previous month. Furthermore, HIV acquisition among MSMW may be from their female sexual partners through unprotected vaginal and anal sex as this study observed that anal sex with female partners is common and condom use is limited. It may be that MSMW engage in sex with female partners with higher risk of HIV; or that MSMW may access MSM‐friendly prevention services less often than MSMO. Further studies, including those leveraging phylogenetic data, are needed to help understand HIV transmission dynamics in different sexual networks. There is a demonstrated need to reinforce current prevention strategies for MSM in Rwanda and implement and scale up HIV Pre‐Exposure Prophylaxis (PrEP) given the low CCU, multiple sexual partnerships, high prevalence of STIs and the prevalence of viraemia found among MSM and TGW in this study.

The association of circumcision with lower prevalence of HIV infection is consistent with findings from a recent systematic review and meta‐analysis showing that circumcision was associated with 42% decreased odds of HIV infection among MSM in low‐ and middle‐income countries (35). This suggests that voluntary medical male circumcision (VMMC) could be an additional prevention strategy for MSM as well (36). In Rwanda, VMMC is currently implemented for prevention of HIV among men and MSM can benefit from this prevention tool.

In this study, one in six participants self‐identified as transgender or a woman, showing that transgender individuals constitute a large portion of the MSM/TGW population in Kigali. This proportion of TGW is comparable to findings from other African countries (7). Consistent with findings worldwide (7, 37, 38), TGW in Kigali were more likely to engage in sex work and experience more stigma and mental health issues compared to cis‐MSM. These structural factors are known to potentiate the risk of HIV acquisition by preventing access to HIV prevention and treatment services (3, 20). Structural interventions including stigma and violence mitigation interventions may support HIV programming for MSM/TGW in Rwanda. The lack of association of gender identity with HIV and STIs may be explained by the small number of transgender individuals in the study resulting in limited power to detect differences. However, the differences found on known HIV risk factors suggest they may have a higher risk of HIV acquisition and urge to consider their needs in HIV programming in Rwanda. A larger study focused on TGW in Rwanda could help understand the epidemiology of HIV in this group.

Among participants living with HIV, under 60% were virally suppressed suggesting suboptimal ART linkage, retention and adherence; as well as potential acquired and transmitted ART resistance. The high proportion of viral suppression among MSM who reported not knowing their status or using ART, suggests that more MSM participants knew their HIV status and were on ART than those who self‐reported. This misreporting of HIV status and engagement in care has been described previously (10). Although, there is a possibility that some are elite controllers, this proportion is much higher than what would be expected based on several studies of elite controllers in Africa (39, 40, 41). Thus, although bio‐behavioural surveys are sometimes the only way to assess MSM and other KPs engagement in care due to the difficulty of identifying them in routine HIV programme data (42, 43), these data reiterate the shortcomings of relying on self‐reported data to estimate HIV status and ART coverage and the potential utility of testing for plasma ART levels in addition to viral load (44). Despite the misclassification, the high proportion of participants living with HIV who were not virally suppressed is evidence of gaps in the first two UNAIDS targets. Consequently, promotion and implementation of novel HIV testing strategies including self‐testing could improve identification of MSM living with HIV and their linkage to care. Furthermore, given that a quarter of participants reporting ART use were viraemic, HIV drug resistance testing and adherence programmes could improve quality of care. Finally, longitudinal studies are needed to understand the implication of these findings on HIV incidence among MSM in Kigali.

This study has several limitations. The cross‐sectional nature of the study limits the ability to make any temporal relationships between HIV and the determinants of interest. The small sample of TGW within the study was underpowered to conduct multivariable analyses stratified by gender identity. The RDS‐II estimator relies on individual reported network size to calculate sampling weights that are used to estimate population estimates. Thus, any biases in reporting individuals’ network size would have introduced bias in the RDS adjusted analyses. The sensitive nature of questions assessing HIV infection and/or risk makes it subject to social‐desirability bias. This limited the ability to precisely estimate the proportion of MSM who knew their HIV status and who were engaged in care. There is also potential for recall bias for questions asking about longer time frames including those on condom use and number and type of partners. However, in this case, the risk is that the data reported are an underestimate of the actual numbers. Finally, restricting recruitment to Kigali city makes the findings less generalizable to other areas of the country.

## 5 CONCLUSION

These data reinforce that MSM/TGW are at high risk for HIV and are currently underserved by HIV prevention and treatment services in Rwanda. Unmet HIV prevention needs included high STI prevalence, limited consistent use of condoms and of condom compatible lubricants. From the perspective of treatment, the level of viral load suppression was below the third target of UNAIDS 90‐90‐90 suggesting opportunities for optimization of linkage and retention services. Moreover, the disconnect between reported treatment status and viral suppression may suggest poor adherence and/or significant primary and secondary ART resistance though this was not assessed. These data suggest the utility of novel HIV treatment and prevention services including decentralized HIV testing strategies and pre‐exposure prophylaxis for MSM/TGW in Rwanda.

## COMPETING INTERESTS

The authors declare no competing interests to disclose.

## AUTHORS’ CONTRIBUTIONS

SDB, SA, EK, PS, AK, SN and PM conceived and designed the study. JOTR, BL, JN, SH, AM and CEL oversaw implementation and data collection. JOTR, OO and SK analysed the data. JOTR wrote the first draft of the paper. All authors reviewed, edited and approved the manuscript.

## ABBREVIATIONS

ART, Antiretroviral treatment; CCU, Consistent Condom Use; CT, Chlamydia Trachomatis; ELISA, Enzyme‐linked Immunosorbent Assay; Frw, Francs Rwandais; HDI, Health Development Initiative; HIV, Human Immunodeficiency virus; IQR, Interquartile range; MSM, men who have sex with men; MSMW, men who have sex with men and women; NG, Neisseria Gonorrhoea; NRL, National reference Laboratory of Rwanda; PrEP, Pre‐exposure Prophylaxis; PSF, Projet San Francisco; RDS, Respondent‐Driven Sampling; RPR, Rapid Plasma Reagin; SSA, sub‐Saharan Africa; STI, Sexually Transmitted Infection; TGW, Transgender women; TPHA, Treponema Pallidum Haemagglutination Assay; UNAIDS, Joint United Nations Programme on HIV/AIDS.

## Supporting information


**Table S1.** Study inclusion criteria
**Table S2.** Items used to assess stigma experiences among men who have sex with men and transgender women in Kigali, Rwanda
**Figure S1.** Individual and structural determinants hypothesized to be associated with HIV infection among men who have sex with men and transgender women in Kigali, Rwanda.
**Table S3.** Comparison of cis‐gender MSM and transgender women on key sociodemographic, biological, behavioral, and HIV/STI outcomes Kigali, Rwanda, 2018.
