# Supplementary material for: HIV infection and engagement in HIV care cascade among men who have sex with men and transgender women in Kigali, Rwanda: a cross‐sectional study
Source: J Int AIDS Soc. 2020 Oct 1;23(Suppl 6):e25604. doi: 10.1002/jia2.25604 (PMC7527755; doi:10.1002/jia2.25604)
Supplement: Supplementary file 1 — Table S1. Study inclusion criteria Table S2. Items used to assess stigma experiences among men who have sex with men and transgender women in Kigali, Rwanda Figure S1. Individual and structural determinants hypothesized to be associated with HIV infection among men who have sex with men and transgender women in Kigali, Rwanda. Table S3. Comparison of cis‐gender MSM and transgender women on key sociodemographic, biological, behavioral, and HIV/STI outcomes Kigali, Rwanda, 2018. [file JIA2-23-e25604-s001.docx]

**Supplemental file: HIV infection and engagement in HIV Care Cascade among Men who have Sex with Men and Transgender Women in Kigali, Rwanda: A cross-sectional study**

**Authors**

Jean Olivier Twahirwa Rwema, Benjamin Liestman, Julien Nyombayire, Sara Herbst, Sosthenes Ketende, Amelia Mazzei, Carrie E. Lyons, Oluwasolape Olawore, Sabin Nsanzimana, Placidie Mugwaneza, Aflodis Kagaba, Patrick Sullivan, Susan Allen, Etienne Karita, Stefan D. Baral

**Study site**

The study site was chosen to mitigate any negative consequences of study participation and to ensure anonymity and privacy. Community members of men who have sex with men (MSM) and transgender women (TGW) organizations participated in the process of identifying the study site.

**Recruitment and eligibility screening**

Two seeds were first enlisted to initiate recruitment and a third seed was enlisted during the study to maximize recruitment among older network of MSM and TGW in Kigali, Rwanda. Sociodemographic characteristics were used in the selection of seeds to maximize sample heterogeneity. The first seed was in the mid-thirties and resided in the rural areas of Kigali city. The second was in the twenties and resided in the center of the city. The third was in the late fifties and resided in Kigali city as well. Recruitment reached 31 of the 35 sectors in Kigali city.

Before any study procedures, participants underwent an eligibility screening interview conducted by trained nurse counselors. A participant had to fulfill all the eligibility criteria to be eligible for the study. The questionnaire included the following questions:

**Supplemental table 1. Inclusion criteria**

| **Eligibility screening questions** |
| --- |
| Was the participant born biologically male ? |
| Has the participant had anal sex with a man in the past 12 months ? |
| Is the participant at least 18 years old? |
| Has the participant lived in Kigali for the past 3 months? |
| Is the participant mentally sound and not under the influence of drugs or alcohol ? |
| Has the participant provided their consent to participate in the behavioral questionnaire ? |
| Has the participant given their biological samples? (Blood, urine, rectal swab) |

Overall, 889 participants presented at the study site and 157 were not eligible participants overall. The majority 91% (143/157) were not eligible because they reported no anal sex with a man in the previous 12 months.

**Analytical framework.**

**Supplemental figure 1.** List of individual and structural determinants hypothesized to be associated with HIV infection among men who have sex with men and transgender women in Kigali, Rwanda

The conceptual framework used for this study was based on a modified social ecological model (MSEM) for HIV risk including individual, network, community, public policy, and HIV-prevalence levels (1). This framework published by Baral et. al, guided the development of the structured interview instrument used to collect data for this study. This framework facilitates the contextualization of individual-level behaviors with network and community-level determinants of risk among MSM. Individual-level biological and behavioral risks among key populations are contextualized by higher order risk factors including size and density of social and sexual networks, as well as stigma operating at the community level and affecting public policies. Studies have consistently demonstrated that the manifestations of stigma targeting key populations in the Sub-Saharan African context limit both the provision and uptake of effective HIV prevention, treatment, and care programs. This model informed the data collection and the analytical approach for this study.

We hypothesized that prevalent HIV infection would be positively associated with greater age, having a higher number of sexual partners, non-consistent condom use, prevalent sexually transmitted infections, identifying as a transgender, engagement in sex work, hazardous alcohol use and depressive symptoms. Furthermore, we expected the different types of stigmas and difficult access to condoms to be positively associated with HIV infection. Factors that were expected to be negatively associated with HIV infection were being circumcised, higher income and higher education level.

**Stigma experiences**

These stigma items used in this study have been used in other studies across sub-Saharan Africa. The questions are table 2Answer options for each question were as follow: ***0=No, 1=Yes, in the last 6 months 2=Yes, but not in the last 6 months, 88=Refusal and 99=Don't know.***

**Supplemental table 2. Items used to assess stigma experiences among men who have sex with men and transgender women in Kigali, Rwanda**

| **Item** | **Stigma scale** |
| --- | --- |
| Have you ever felt excluded from family activities because you have sex with men? | Perceived |
| Have you ever felt that family members have made discriminatory remarks or gossiped about you because you have sex with men? | Perceived |
| Have you ever felt rejected by your friends because you have sex with men? | Perceived |
| Have you ever felt afraid to go to health care services because you worry someone may learn you have sex with men? | Anticipated |
| Have you ever avoided going to health care services because you worry someone may learn you have sex with men? | Anticipated |
| Have you ever felt that you were not treated well in a health center because someone knew that you have sex with men? | Perceived |
| Have you ever heard health care providers gossiping about you (talking about you) because you have sex with men? | Enacted |
| Have you ever felt that the police refused to protect you because you have sex with men? | Perceived |
| Have you ever felt scared to be in public places because you have sex with men? | Anticipated |
| Have you ever been verbally harassed and felt it was because you have sex with men? | Enacted |
| Have you ever been blackmailed by someone because you have sex with men? | Enacted |
| Has someone ever physically hurt you (pushed, shoved, slapped, hit, kicked, choked, or otherwise physically hurt you)? Do you believe any of these experiences of physical violence was/were related to the fact that you have sex with men? | Enacted |
| Have you ever been forced to have sex when you did not want to? (By forced, I mean physically forced, coerced to have sex, or penetrated with an object, when you did not want to). Do you believe any of these experiences of sexual violence were related to the fact that you have sex with men? | Enacted |

During analyses, the items were dichotomized into 0: No and 1: Yes, and refusal and do not know were considered as missing. Given that the proportion of missing values were very small for all items (<1%), no imputations were necessary. We then created three different stigma scales: perceived, anticipated, and enacted stigma. For each score, an individual score was calculated by summing up individual responses on each question of that scale using 0 for a negative answer and 1 for a positive answer. Scores ranged from 0 to 5 for perceived stigma, 0 to 3 for anticipated stigma and 0 to 5 for enacted stigma. These scores were then dichotomized to 1 and a score of 1 or above was considered as experiencing that type of stigma. The results are reported in the results section and tables 2&3 of the manuscript.

**Results**

**Supplemental table 3. Comparison of cis-gender MSM and transgender women on key sociodemographic, biological, behavioral, and HIV/STI outcomes Kigali, Rwanda 2018**

|  | **Cis-gender % (N)** | **Transgender % (N)** | **P value** |
| --- | --- | --- | --- |
| **Sociodemographic characteristics** | | | |
| **Age in years** |  |  |  |
| 18-24 | 46.6 (294) | 39.6 (42) | **0.038** |
| 25-34 | 38.4 (242) | 50.9 (54) |  |
| Over 35 | 15.1 (95) | 9.5 (10) |  |
| **Education** |  |  |  |
| Primary level or less | 29.2 (184) | 24.5 (26) | 0.452 |
| Some secondary | 33.4 (211) | 32.1 (34) |  |
| Secondary or above | 37.4 (236) | 43.4 (46) |  |
| **Marital status** |  |  |  |
| Single/ Never married | 88.3 (557) | 91.5 (97) | **0.075** |
| Cohabitating with male partner | 3.3 (21) | 5.7 (6) |  |
| Cohabitating/married with female partner | 3.3 (21) | 2.8 (3) |  |
| Divorced/Separated/Widow | 5.1 (32) | 0 (0) |  |
| **Monthly Income (Frw)** |  |  |  |
| Less than 50,000 | 66.0 (416) | 62.3 (66) | 0.45 |
| Over 50,000 | 34.0 (214) | 37.7 (40) |  |
| **Self-reported sexual preference** |  |  |  |
| Gay or Homosexual | 60.4 (381) | 89.6 (95) | **0.0001** |
| Bisexual | 34.4 (217) | 9.4 (10) |  |
| Heterosexual | 5.2 (33) | 1.0 (1) |  |
| **Biological** | | | |
| **Depression** |  |  |  |
| No depression | 68.2 (430) | 44.3 (47) | **0.0001** |
| Mild depressive symptoms | 23.9 (151) | 40.6 (43) |  |
| Moderate depressive symptoms | 5.6 (35) | 12.3 (13) |  |
| Moderately severe depressive symptoms | 1.7 (11) | 0.9 (1) |  |
| Severe depressive symptoms | 0.6 (4) | 1.9 (2) |  |
| **Circumcision** |  |  |  |
| No | 22.8 (144) | 16.1 (17) | 0.118 |
| Yes | 77.2 (487) | 83.9 (89) |  |
| **Behavioral** | | | |
| **Age of first sex with male** |  |  |  |
| Before 19 years | 51.4 (324) | 67.9 (72) | **0.005** |
| 19 to 22 years | 23.3 (147) | 17.9 (19) |  |
| Over 22 years | 25.3 (159) | 14.2 (15) |  |
| **CCU with male partners in the last 6 months** |  |  |  |
| Non consistent condom use | 87.0 (549) | 88.7 (94) | 0.633 |
| Consistent condom use | 13.0 (82) | 11.3 (12) |  |
| **CCU at most recent sex with male partners** |  |  |  |
| Non consistent condom use | 86.7 (547) | 81.1 (86) | 0.128 |
| Consistent condom use | 13.3 (84) | 18.9 (20) |  |
| **Lubricant use** |  |  |  |
| Never | 14.3 (90_ | 4.7 (5) | **0.007** |
| Ever used lubricant | 85.7 (541) | 95.3 (101) |  |
| **Number of regular sexual partners in the last month** |  |  |  |
| None | 43.9 (277) | 31.1 (33) | 0.105 |
| 0ne to two | 35.6 (224) | 43.4 (46) |  |
| Two to three | 9.7 (61) | 12.3 (13) |  |
| Over three | 10.8 (68) | 13.2 (14) |  |
| **Number of casual sexual partners in the last month** |  |  |  |
| None | 52.9 (333) | 44.8 (47) | 0.306 |
| One to three | 34.8 (219) | 40.9 (43) |  |
| Over three | 12.3 (78) | 14.3 (15) |  |
| **Sex with women** |  |  |  |
| Never | 22.1 (139) | 45.3 (48) | 0.0001 |
| Yes, but not in the last 12 months | 37.9 (239) | 37.7 (40) |  |
| Yes, in the last 12 months | 40 (252) | 17.0 (18) |  |
| **Professional sex worker in the last 12 months** |  |  |  |
| No | 86.8 (548) | 72.6 (77) | **0.0001** |
| Yes | 13.2 (83) | 27.4 (29) |  |
| **Alcohol use** |  |  |  |
| Nonhazardous alcohol use | 23.6 (149) | 25.5 (27) | 0.678 |
| Hazardous alcohol use | 76.4 (482) | 74.5 (79) |  |
| **Structural** | | | |
| **Anticipated stigma** |  |  |  |
| No | 61.8 (390) | 45.3 (48) | **0.001** |
| Yes | 38.2 (241) | 54.7 (58) |  |
| **Perceived stigma** |  |  |  |
| No | 69.4 (438) | 34.9 (37) | **0.0001** |
| Yes | 30.6 (193) | 65.1 (69) |  |
| **Enacted stigma** |  |  |  |
| Never | 58.6 (370) | 35.8 (38) | **0.0001** |
| Yes | 41.4 (261) | 64.2 (68) |  |
| **HIV and STI related outcomes** | | | |
| Ever tested for HIV |  |  |  |
| Never tested for HIV | 9.2 (58) | 9.4 (10) | 0.917 |
| Ever | 90.8 (572) | 90.6 (96) |  |
| **HIV infection** |  |  |  |
| Negative | 89.8 (566) | 90.6 (96) | 0.818 |
| Positive | 10.2 (64) | 9.4 (10) |  |
| **Virally suppressed (based on <200 copies/ml)** |  |  |  |
| No | 36.5 (23) | 60 (6) | 0.158 |
| Yes | 63.5 (40) | 40 (4) |  |
| **STI diagnosis** |  |  |  |
| Negative | 80.2 (506) | 79.3 (84) | 0.822 |
| Positive | 19.8 (125) | 20.7 (22) |  |
| **Gonorrhea** |  |  |  |
| Negative | 91.6 (576) | 88.7 (94) | 0.332 |
| Positive | 8.4 (53) | 11.3 (12) |  |
| **Chlamydia** |  |  |  |
| Negative | 91.3 (574) | 88.7 (94 | 0.394 |
| Positive | 8.7 (55) | 11.3 (12) |  |
| **Syphilis** |  |  |  |
| Negative | 94.0 (592) | 96.2 (102) | 0.354 |
| Positive | 6.0 (38) | 3.8 (4) |  |

**References**

1. Baral S, Logie CH, Grosso A, Wirtz AL, Beyrer C. Modified social ecological model: a tool to guide the assessment of the risks and risk contexts of HIV epidemics. BMC Public Health. 2013;13(1):482-.
